# Association between arterial stiffness and left ventricular diastolic function in relation to gender and age

**DOI:** 10.1097/MD.0000000000005783

**Published:** 2017-01-10

**Authors:** Hack-Lyoung Kim, Woo-Hyun Lim, Jae-Bin Seo, Woo-Young Chung, Sang-Hyun Kim, Myung-A. Kim, Joo-Hee Zo

**Affiliations:** Division of Cardiology, Department of Internal Medicine, Boramae Medical Center, Seoul National University College of Medicine, Seoul, Korea.

**Keywords:** age, arterial stiffness, diastolic function, gender, pulse wave velocity

## Abstract

Left ventricular (LV) diastolic dysfunction and subsequent overt heart failure are more prevalent in elderly women. Close interaction between arterial stiffness and LV morphology/function has been reported. The aim of this study was to investigate whether there is an age- and gender-dependent relationship between arterial stiffness and LV diastolic function. A total of 819 subjects (58.6 ± 13.3 years, 50.2% men) without structural heart disease (LV ejection fraction ≥50%) were retrospectively analyzed. All participants underwent transthoracic echocardiography and brachial-ankle pulse wave velocity (baPWV) measurement on the same day. The association of baPWV with septal e′ velocity and average E/e′ was assessed. In the total study subjects, baPWV was negatively correlated with septal e′ velocity (*r* = 0.383, *P* < 0.001), and positively correlated with E/e′ (*r* = −0.266, *P* < 0.001). These linear correlations remained significant even after stratificaion of the study subjects by age (<65 years vs ≥65 years) and genders (*P* < 0.05 for each). There were obvious differences in baPWV according to groups with normal LV diastolic function, intermediate profile and LV diastolic dysfunction in young (*P* = 0.010) and elderly (≥65 years) women (*P* < 0.001) and eldery men (*P* = 0.012) but not in elderly men (*P* = 0.270). There was a significant association of baPWV with septal e′ velocity (*β* = −0.258, *P* = 0.020) and E/e′ (*β* = 0.122, *P* = 0.030) in elderly women even after controlling for multiple clinical covariates. This independent association was not seen in younger women and men (*P* > 0.05 for each). In conclusion, baPWV was independently associated with septal e′ velocity and E/e′ in elderly women but not in younger women or men. The results of this study provide additional evidence that increased arterial stiffness plays an important role in the development of heart failure with preserved ejection fraction as well as LV diastolic dysfunction in elderly women.

## 1 INTRODUCTION

Arterial stiffens with aging and arteriosclerosis of arterial walls.^[1]^ Information on arterial stiffness is valuable because arterial stiffness has been proposed as an independent risk factor for future cardiovascular events.^[2–5]^ Pulse wave velocity (PWV) is simple, most validated, and widely used to measure arterial stiffness in research and clinical fields.^[6]^ Body of evidence from recent studies has shown the imporant role of arterial stiffness on left ventricular (LV) diastolic function.^[7–10]^ Increased arterial stiffness may promote LV hypertrophy and reduce coronary perfusion, which seem to be related to LV diastolic dysfunction and heart failure with preserved ejection fraction (HFpEF).^[10,11]^ Although it has been demonstrated that HFpEF is more prevalent in elederly women,^[12,13]^ its underlying pathophysiology has not yet been fully understood. Recent studies have suggested that increased arterial stiffness is an important contributor to the development of heart failure.^[11,14]^ It has been demonstrated that hemodynamics of the arterial system is different according to gender and age^[15]^: greater arterial stiffness and higher vascular loading have been described in elderly woman, compared to young women and age-matched men.^[16,17]^ However, age- and gender-related differences in the association between arterial stiffness and LV diastolic function are still conflicting. Additionaly, comprehensive analysis of the interplay between age and gender on this issue has been scarce.^[5]^ Therefore, this study was performed to investigate age and gender related differences in the association between LV diastolic function and arterial stiffness. Our hypothesis was that the association between arterial stiffness and LV diastolic funcion could be more pronounced in elderly women than in young women or men.

## 2 METHODS

### 2.1 Study subjects

This single center study was performed at Boramae Medical Center (Seoul, Korea). We retrospectively reviewed a total of 925 subjects without overt cardiovascular disease undergoing brachial-ankle PWV (baPWV) measurement and transthoracic echocardiography (TTE) as their routine check-up between August 2013 and July 2014. In this study, baPWV measurement and TTE were perfomred on the same day. We excluded subejcts with the following conditions: history of coronary artery disease (coronary revascularization with percutanous coronary intervention or coronary bypass surgery and myocardial infarction) (n = 35), nonsinus rhythm (n = 21), low ankle-brachial index (<0.9) (n = 12), low left eventricular systolic function (LV ejection fraction [EF] < 50%) (n = 12), regional wall motion abnoriality (n = 10), valvular stenosis or regurgitation more than mild degree (n = 9), and pericardial effusion (n = 7). Finally, 819 subjects were analyzed in this study. All study subjects were in medically stable conditions at the time of health check-up. Height and weight were measured, and body mass index (BMI) was calculated as a subject's weight in kilogram divided by the square of hieght in meters. Systolic and diastolic blood pressures and heart rate were measured on the right upper arm by a trained nurse using an oscillometric device. Information on underlying medical illness including hypertension, diabetes melliuts, and dyslipidemia was obtained by using a standarized questionnaire, and maintainance medications or laboraory test results were indentified. Hypertension was defined as history of hypertension, antihypertensive medications, or repeated measurements of systolic blood pressure ≥140 mm Hg or diastolic blood pressure ≥90 mm Hg. Diabetes mellitus was defined as history of diabetes mellitus, antidiabetic medications, or fasting glucose ≥126 mg/dL at 2 or more measurements. Dyslipidemia was defined as history of dyslipidemia, lipid-lowering medication, or a pretesttotal cholesterol of ≥200 mg/dL or a low-density lipoprotein (LDL) cholesterol of ≥160 mg/dL. Information on smoking habits was also obtained, and subjects who smoked regularly during the previous 12 months were classified as smokers. All subjects underwent laboratory tests by sampling venous blood in the morning after overnight fasting. White blood cell count (WBC), hemoglobin, concentration and serum levels of fasting glucose, total cholesterol, LDL cholesterol, high-density lipoprotein (HDL) cholesterol, triglyceride, creatinine, and C-reactive protein were measured by an automated enzymatic procedure. Estimated glomerular filtration rate (eGFR) was calculated using the 4-component MDRD (Modification of Diet in Renal Disease) equation incorporating age, race, sex, and serum creatinine level.^[18]^ The Institutional Review Board of Boramae Medical Center (Seoul, Korea) approved this study protocol. Informed consent was waived due to the routine nature of information collected and retrospective study design.

### 2.2 baPWV measurement

In this study, baPWV measurements were performed between 9:00 and 11:00 in the morning. Subjects were studied in the supine position after approximately 5 minutes of rest in an atemperature-controlled and quiet environment. Patients were allowed to take their regular medications, but, smoking, alcohol drinking, and caffein consumption were prohibited on the day of examination. The baPWV values were measured noninvasively using an automated waveformanalyzer (VP-1000; Colin Co. Ltd, Komaki, Japan).^[19]^ Briefly, arterial pulse wave was measured on patients’ bilateral upper (brachial artery) and lower (posterior tibialartery) extremities with gentle pressure using applanation tonometry, which simultaneously recorded blood pressures, electrocardiograms, and heart sounds in accordance with the manufacturer's recommendations. The baPWV values were calculated as distance between the brachial and posteriortibial arteries divided by time interval (velocity = distance/time) (cm/s). The distance between the brachialis and posterior tibial arteries was estimated on the basis of the height of the subject, and time interval between them was calculated by the time difference between waves in front of them. The average value between the left and right baPWV measurements was chosen for all analyses. All measurements were made by the same experienced operator blinded to all clinical data. The intraobserver coefficient of variation for baPWV was 5.1% in our laboratory.^[20]^

### 2.3 TTE

TTE was performed by experienced cardiologists or cardiosonographers using a 2.5-MHz probe with commercially available ultrasound systems (iE33, Philips, Andover, MA, USA; or Vivid E9, GE Medical, Milwaukee, WI, USA) according to the standardized protocol. M-mode images from the parasternal short-axis view were obtained to measure LV internal dimension during systole (LVIDs) and diastole (LVIDd), LV septal wall thickness during diastole (SWTd), and LV posterior wall thickness during diastole (PWTd). LV mass was calculated using the following formula: LV mass = 0.8 × {1.04 × [(LVIDd + PWTd + SWTd)^3^ − LVIDd^3^]} + 0.6 g, and indexed for body surface area (= LV mass index [LVMI]).^[21]^ Simpson biplane method was employed in the calculation of LVEF. Early peak transmitral filling velocities during early diastole (E) was imaged at the tip of the mitral leaflets from the apical 4-chamber view. Color-coded tissue Doppler imaging (TDI) was applied to the apical 4-chamber view to determine peak early (e′) velocities at both septal and lateral annuli. The average value of septal and lateral e′ velocities was used for E/e′, which was calculated to estimate LV filling pressure. Peak velocity through tricuspid regurgitation was obtained in modified 4-chamber view. The left atrial volume (LAV) was calculated using the biplane method and indexed to the body surface area (LAV index, LAVI).^[21]^ Recent guidelines emphasize the important of role of septal e′ velocity and average E/e′ in assessing LV diastolic function in subjects with normal LVEF.^[22]^ Our study focused on these 2 TDI-derived diastolic indices. According to the guideline's recommendations,^[22]^ we considered LV diastolic function normal if none or one available parameter met cut-off values (septal e′ velocity >7 cm/s, average E/e′ >14, tricuspid regurgitation velocity >2.8 m/s, and LAVI >34 mL/m^2^). LV diastolic dysfunction was considered intermediate in cases meeting 2 cut-off values. If more than 2 parameters met cut-off values, a diagnosis of LV diastolic dysfunction was made. The interobserver coefficients of variation for e′ and E/e′ were 6.8% and 7.0%, respectively, in our laboratory.

### 2.4 Statistical analysis

Continuous variables are expressed as mean ± standard deviation, whereas categorical variables are presented as absolute values and their proportions. Continuous variables were compared using Student *t* test, and categorical variables were compared using the Chi-square test between the 2 groups. Pearson bivariate correlation analyses were used to investigate a linear association between baPWV and diastolic indices, and the linear correlations were demonstrated using scatter plots. Analysis of variance (ANOVA) was used in comparisons of mean baPWV values according to the LV diastolic function status (normal, intermediate, and diastolic dysfunction). Multiple linear regression analyses were performed to investigate the independent association between baPWV and diastolic indices. Age, BMI, systolic blood pressure, heart rate, fasting glucose, total cholesterol, and eGFR were considered potential confounders and adjusted in multivariable models. A *P*-value of <0.05 was considered significant. All analyses were performed using SPSS 18.0 statistical package (IBM Co., Armonk, NY).

## 3 RESULTS

### 3.1 Clinical characteristics of the study subjects

The clinical characteristics of the study subjects accroding to age (<65 years vs ≥65 years) and gender are shown in Table 1. Elderly (age ≥65 years) men had a lower BMI than youger (age <65 years) men, whereas elderly women had a higher BMI than younger women. Systolic blood pressure was elevated in elderly subjects than in youger subjects in both genders. Among traditional cardiovascular risk factors, the incidence of diabetes mellitus was higher in elderly women than in youger women. More men had smoked in the young age goup than in the old age group. The blood hemoglobin concentrations and cholesterol levels and renal function were lower in elderly subjects than in youger subjects in both genders. LV mass was increased and LV diastolic function was worsened in elederly subjects than in younger subjects in both genders. The baPWV values were signinficantly higher in elderly subjects than in younger subjects in both genders.

**Table 1:**
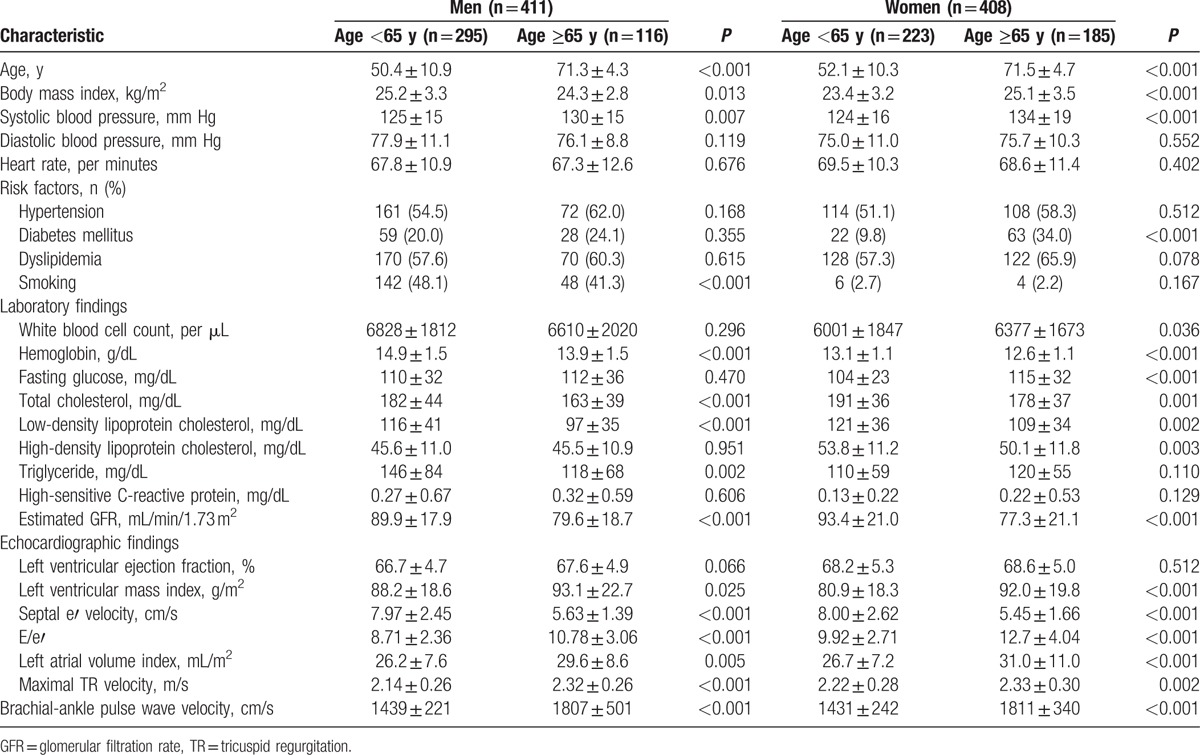
Clinical characteristics of study subjects according to gender and age.

### 3.2 Association between baPWV and LV diastolic function

**Figure 1:**
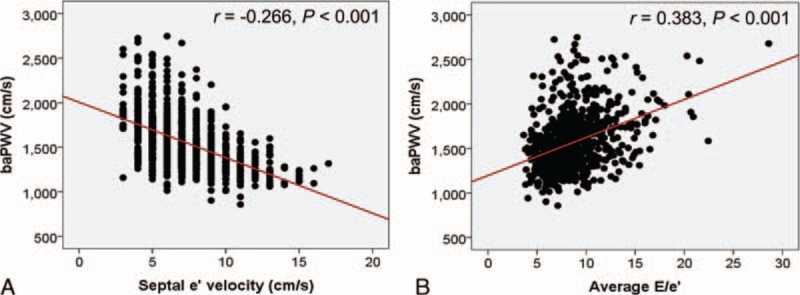
Linear correlations of baPWV with septal e′ velocity (A) and average E/e′ (B). baPWV = brachial-ankle pulse wave velocity.

**Figure 2:**
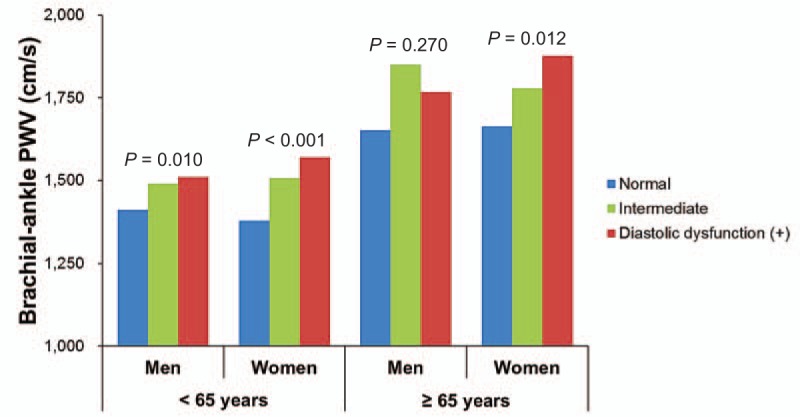
Brachial-ankle PWV and left ventricular diastolic function in relation to gender and age. PWV = pulse wave velocity.

In the total study subjects, baPWV was negatively correlated with septal e′ velocity (*r* = −0.266, *P* < 0.001), and positively correlated with E/e′ (*r* = 0.383, *P* < 0.001) (Fig. 1). These linear correlations remained even after stratificaion of the study subjects by age and genders (*P* < 0.05 for each) (Table 2). Ovious differences in baPWV were found between the groups with normal LV diastolic function, intermediate profile and LV diastolic dysfunction in young (ANOVA *P* = 0.010) and elderly women (ANOVA *P* < 0.001) and eldery men (ANOVA *P* = 0.012) but not in elderly men (ANOVA *P* = 0.270) (Fig. 2). In elderly women, baPWV had significant association with septal e′ velocity (*β* = −0.258, *P* = 0.020) and E/e′ (*β* = 0.122, *P* = 0.030) even after controlling for multiple clinical corvariates. However, no statistical significance was observed in young women and men in the same multivariable analyses (*P* > 0.05 in for each model) (Table 3).

**Table 2:**
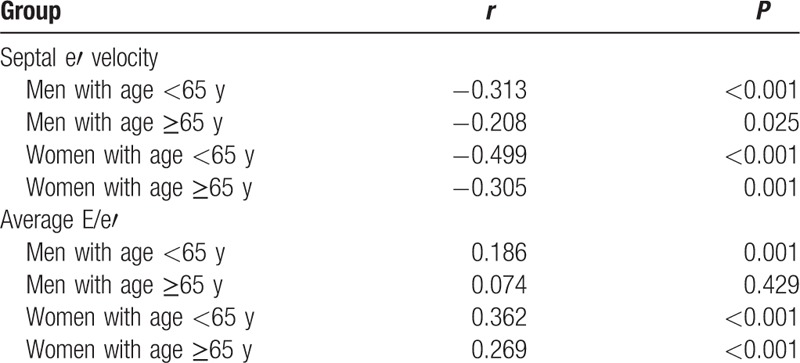
Univariate linear associations between brachial-ankle pulse wave velocity and major 2 diastolic indices in each gender and age group.

**Table 3:**
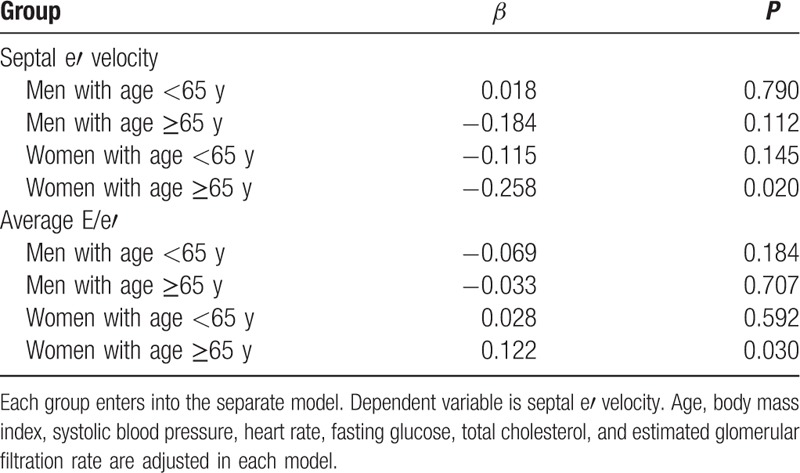
Independent association between brachial-ankle pulse wave velocity and 2 major diastolic indices in each gender and age group.

## 4 DISCUSSION

In a healthy cohort of middle-aged and elderly individuals without documented cardiovascular disease, we found independent associations of baPWV with LV septal e′ velocity and average E/e′ in elderly women. Although there were linear associations between baPWV and the 2 diastolic indices in univariate analysis, the statistical significance of these associations vanished after multivariable adjustment in youger women or men. This suggests a stronger interplay between LV and the arterial system in eldelry women, and supports the evidence that increased arterial stiffness may be one of the important factors for the development of HFpEF as well as LV diastolic dyfunction in eldery women.

Several prior studies have adressed the association between LV diastolic function and arterial stiffness in normal subjects. Cauwenberghs et al^[7]^ investigated 1233 subjects from the general population, and showed that diastolic parameters are significalty correlated with cfPWV and central pulse pressure as measured by arterial tonometry. A recent large study of 5799 participants (Framingham Heart Study) also showed significant correlations of cfPWV and central pulse pressure with lateral e′ velocity and E/e′.^[8]^ Borlaug et al^[23]^ performed noninvasive measurement using applanation tonometry in 58 participants, and showed that carotid augementation index and carotid characteristic impedence have an independent association with LV septal e′ velocity. Similarly, our results showed that arterial stiffness measured by baPWV was signficantly correlated with septal e′ velocity and E/e′ in univariate anslyses. When we performed multivariable analyses on the total subjects without stratification according to age and gender, there was an independent associaion between arterial stiffness and LV diastolic indicies (data not shown).

The exact pathophysiology underlying the association between LV diastolic function and arterial stiffness has not yet been completely elucidated. However, it has generally been accepted that increased arteiral stiffness influences the development of LV diastolic dysfunction, but not vice versa. Increased afterload by a stiff artery causes LV hypetrophy and a decrease in diastolic pressure associated with reduced coronary flow.^[10,11]^ All these fasctors are main contributors to LV diastolic impairment. A pararell increse in arterial stiffness and LV diastolic dysfunction is also a possible mechanism due to shared risk factors and common pathophysiology of the 2 conditions. Indeed, it has been suggested that patients with greater arterial stiffness are more likely to have increased LV stiffness,^[24,25]^ and that these concurrent increases in ventricular and arterial stiffness with aging have been implicated in the pathogenesis of heart failure.^[26]^ The causal relationship between increased arterial stiffness and LV diastolic dysfunction was not observed in our cross-sectional analysis, which remains to be elucidated with further longitudinal studies.

Gender-related differences in the association between arterial stiffness and LV diastolic dysfunction have been reported; however, results are still debatable. Weber et al^[27]^ found, in a study of 336 patients undergoing coronary angiography, that e′ velocity and LV filling pressure are significantly correlated with augmentation index and PWV, and that these correlations were independent of age and gender. Abhayaratna et al^[28]^ showed, in a study of 233 elderly subjects with normal LVEF, that brachial pulse pressure, central pulse pressure, and PWV progressively increase according to the severity of diastolic dysfunction in both men and women. Russo et al^[17]^ investigated 983 community subjets to assess gender difference, and showed that the stength of the association between arterial stiffness and LV diastolic function is similar betwen men and women even though higher arterial stiffness and more impaired LV diastolic function are observed in women. On the other hand, Shim et al^[14]^ showed, in a study of 158 age-matched subjects without structural heart disease, that LV diastolic function correlated significantly with the parameters representing arterial stiffness in women but not men. Similarly, Redfield et al^[25]^ investigated 2042 participants from the community, and showed tha advanced age and female gender are associated with increased arterial and LV diastolic stiffness. Our results support their studies demonstrating a pronounced association between baPWV and diastolic indices in eldelry women. Different study populations and measurements with different sensitivities for vascular stiffening might be, in part, responsible for different findings among studies.

There are many echocardiographic parameters used to assess LV diastolic function. Among them, TDI-derived parameters have deserved attention. During diastole, e′ velocity is closely related with LV relaxation,^[29]^ and E/e′ has proved to be a useful surrogate measure of LV filling pressure compared to other diastolic indices.^[30]^ Recent guidelines also emphasize the importance and usefullness of these parameters for evaluating LV diastolic function, and recommend initial use of these parameters in the assessment of LV diastolic function especially in subjects with normal LVEF.^[22]^

Our results may highlight the potential contribution of increased pulsatile load and LV-arterial coupling to LV diastolic dysfunciton in elderly women. Given that LV diastolic dysfunction is a main determinant of HFpEF, it can also be postulated that increased arterial stiffness is an important risk factor developing HFpEF in elderly women. In addition, arterial stiffness may be a good monitoring tool to assess LV diastolic dysfunction. More importantly, improving arterial stiffness may be a good therapeutic target for delays in the progression of LV diastolic dysfunction or for the prevention of overt clinical heart failure syndrome, espeically in elderly women. Furthermore, from a practical view point, considering that baPWV is a simple and reliable measure of arterial stiffness,^[31]^ baPWV can be a useful target for the treatment of HFpEF as well as a tool to monitor patients with HFpEF.

Our study results should be interpreted in the context of its potential limitations. First, the cross-sectional study design could not confer the causal relationship between LV diastolic function and arterial stiffness, and how gender modifies the relationship was not elucidated. Further longitudinal studies and mechnism analyses are warranted. Secondly, the impact of medications on the association between arterial stiffness and LV diastolic function was not assessed. Thirdly, the indices of arterial stiffness and LV diastolic function were all noninvasive measures. Invasive parameters, more reliable indicators, may have been valuable in our study. Finally, our study population consisted of Korean adults without documented cardiovascular disese with normal LVEF: thus, direct application of our results to other groups of people may be diffucult.

In the present study, baPWV was independently associated with septal e′ velocity and E/e′ in elderly women with normal LVEF but not in young women or men. Our results provide additional evidence that increased arterial stiffness plays an important role in the development of HFpEF as well as LV diastolic dysfunction in elderly women. Further studies are needed to confirm our findings.
